# Prevalence estimation of significant fibrosis because of NASH in Spain combining transient elastography and histology

**DOI:** 10.1111/liv.15323

**Published:** 2022-06-07

**Authors:** José L. Calleja, Jesús Rivera‐Esteban, Rocío Aller, Marta Hernández‐Conde, Javier Abad, Juan M. Pericàs, Hugo G. Benito, Miguel A. Serra, Amparo Escudero, Javier Ampuero, Ana Lucena, Yolanda Sánchez, María T. Arias‐Loste, Paula Iruzubieta, Manuel Romero‐Gómez, Salvador Augustin, Javier Crespo

**Affiliations:** ^1^ Department of Gastroenterology and Hepatology Hospital Universitario Puerta de Hierro, School of Medicine, Universidad Autónoma Madrid, IDIPHIM Majadahonda Spain; ^2^ Liver Unit Vall d'Hebron Hospital Universitari, Vall d'Hebron Institut de Recerca (VHIR), Vall d'Hebron Barcelona Hospital Campus Barcelona Spain; ^3^ Universitat Autònoma de Barcelona Bellaterra Spain; ^4^ Department of Gastroenterology Clinic University Hospital, Medical School, Group of Biomedical Research in Critical Care Medicine (BioCritic), University of Valladolid Valladolid Spain; ^5^ Research Unit Clinic University Hospital, Medical School, Institute of Health Sciences of Castille and Leon (IECSCYL), Group of Biomedical Research in Critical Care Medicine (BioCritic) Valladolid Spain; ^6^ Digestive Medicine Service Clinic University Hospital, University of Valencia Valencia Spain; ^7^ Digestive Diseases Department and CIBERehd Virgen del Rocío University Hospital, Institute of Biomedicine of Seville, University of Seville Seville Spain; ^8^ Gastroenterology and Hepatology Department Marqués de Valdecilla University Hospital, Clinical and Translational Digestive Research Group, IDIVAL Santander Spain; ^9^ Therapeutic Area Cardio‐Metabolism and Respiratory Medicine Boehringer Ingelheim International GmbH Ingelheim am Rhein Germany

**Keywords:** hepatic fibrosis, liver biopsy, non‐alcoholic steatohepatitis, transient elastography

## Abstract

**Background & Aims:**

Non‐alcoholic fatty liver disease (NAFLD) has become a major public health problem, but the prevalence of fibrosis associated with non‐alcoholic steatohepatitis (NASH) is largely unknown in the general population. This study aimed to provide an updated estimation of the prevalence of NASH fibrosis in Spain.

**Methods:**

This was an observational, retrospective, cross‐sectional, population‐based study with merged data from two Spanish datasets: a large (*N* = 12 246) population‐based cohort (ETHON), including transient elastography (TE) data, and a contemporary multi‐centric biopsy‐proven NASH cohort with paired TE data from tertiary centres (*N* = 501). Prevalence for each NASH fibrosis stage was estimated by crossing TE data from ETHON dataset with histology data from the biopsy‐proven cohort.

**Results:**

From the patients with valid TE in ETHON dataset (*N* = 11 440), 5.61% (95% confidence interval [95% CI]: 2.53‐11.97) had a liver stiffness measurement (LSM) ≥ 8 kPa. The proportion attributable to NAFLD (using clinical variables and Controlled Attenuation Parameter) was 57.3% and thus, the estimated prevalence of population with LSM ≥ 8 kPa because of NAFLD was 3.21% (95% CI 1.13–8.75). In the biopsy‐proven NASH cohort, 389 patients had LSM ≥ 8 kPa. Among these, 37% did not have significant fibrosis (F2‐4). The estimated prevalence of NASH F2‐3 and cirrhosis in Spain's adult population were 1.33% (95% CI 0.29–5.98) and 0.70% (95% CI 0.10–4.95) respectively.

**Conclusions:**

These estimations provide an accurate picture of the current prevalence of NASH‐related fibrosis in Spain and can serve as reference point for dimensioning the therapeutic efforts that will be required as NASH therapies become available.

**Abbreviations**
ALP: alkaline phosphatase
ALT: alanine aminotransferase
AST: aspartate aminotransferase
AUDIT: Alcohol Use Disorders Identification Test
BMI: body mass index
CAP: controlled attenuation parameter
CI: confidence interval.
GGT: gamma‐glutamyl transferase
HBsAg: hepatitis B surface antigen
HCV: hepatitis C virus
HDL: high‐density lipoprotein
INE: Instituto Nacional Estadística
LDL: low‐density lipoprotein
LSM: liver stiffness measurement
NAFLD: Non‐alcoholic fatty liver disease
NASH: non‐alcoholic steatohepatitis
NCEP‐ATP III: National Cholesterol Education Program‐Adult Treatment Panel III
TE: transient elastography

**Lay summary**

Non‐alcoholic steatohepatitis (NASH) has become a major public health issue worldwide, but the exact prevalence in the general population of the different stages of liver fibrosis associated to NASH is largely unknown. In the present study, we merged data from a large general population‐based dataset and a contemporary multicentric biopsy‐proven NASH cohort to provide updated prevalence estimates for NASH fibrosis in Spain. These estimates might be leveraged for designing future interventions for NASH.

## 1 INTRODUCTION

Non‐alcoholic fatty liver disease (NAFLD) has become a major public health problem. Both its prevalence and incidence have risen sharply in the last decades.^1^, ^2^, ^3^, ^4^, ^5^ and, by 2030, cirrhosis and hepatocellular carcinoma because of NAFLD are expected to increase further worldwide.^6^, ^7^, ^8^ This threat is aggravated by the fact that there are currently no approved pharmacologic therapies for non‐alcoholic steatohepatitis (NASH). Accurate and geographically‐specific estimations of NASH prevalence by fibrosis stage are paramount for the design and implementation of public health measures and therapeutic strategies.

Nonetheless, the exact prevalence of NAFLD and for the different fibrosis stages of NASH remains unknown to date because previous calculations are subject to various problems. First, reports on NAFLD prevalence base their predictions on non‐invasive diagnostic tests,^9^, ^10^, ^11^, ^12^, ^13^ registry diagnostic codes^14^ or on indirect extrapolations from basic metabolic demographics.^15^ All these methods have suboptimal sensitivity and probably underestimate the real prevalence of NAFLD.^2^, ^16^ In recent years, Controlled Attenuation Parameter (CAP) by transient elastography (TE) has been proven more sensitive for the detection of steatosis than ultrasound or serum‐based scores^10^, ^17^, ^18^, ^19^, ^20^ but reports on NAFLD at the general population level using CAP are still scarce and small‐scale.^21^ Second, estimations of NASH at population level have been extrapolated from either small autopsy or living‐donor series^22^ or through back‐calculations crossing population‐scale estimates for NAFLD with non‐contemporary NASH biopsy series^23^, ^24^, ^25^ subject to selection and ascertainment biases, leading to overrepresentation of more advanced stages of NASH. An intermediate approach consists of the use non‐invasive tests for estimations of liver fibrosis (with transient elastography and/or serum scores) as proxy for NASH in different population‐based studies.^26^, ^27^ However, stratification of patients by fibrosis stage with these methods has been proven suboptimal and histological confirmation of fibrosis predictions is scarce.^9^, ^10^ Finally, the attribution of NAFLD causality in those fibrosis‐based population studies has been indirect (basically based on comorbidities, without concurrent measurement of hepatic steatosis).

The aim of the present study was to provide an updated, accurate, real‐life estimate of the prevalence of NASH‐related fibrosis in Spain. For that purpose, we merged data from a large study on the use of TE for screening of liver disease in the general population of Spain with contemporary data from a multi‐centre cohort of biopsy‐proven NASH from real practice from our country.

## 2 METHODS

### 2.1 Aim and study design

This was an observational, retrospective, cross‐sectional, population‐based, epidemiological study. The main objective of the study was to provide updated estimates for the prevalence of NASH‐related fibrosis in the general population in Spain, with a special focus on those stages at higher risk of complications and that would be eventually amenable to receive pharmacologic therapy under the current regulatory framework, that is F2‐4 fibrosis stages.

**FIGURE 1:**
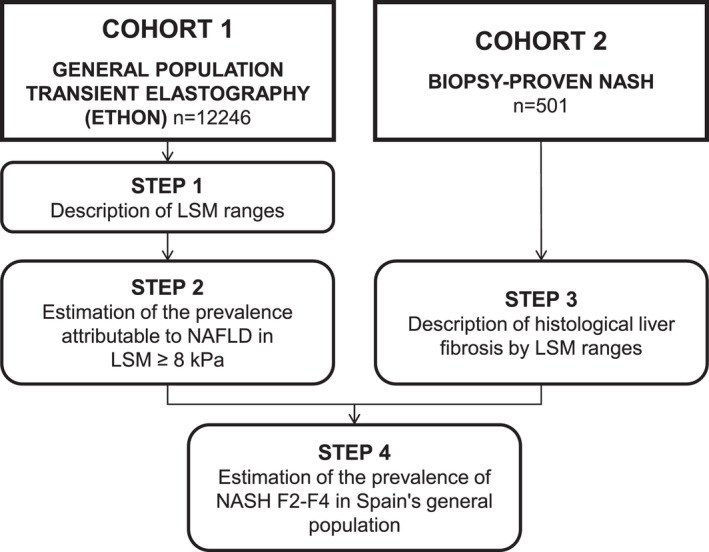
Study design and cohorts.

**FIGURE 2:**
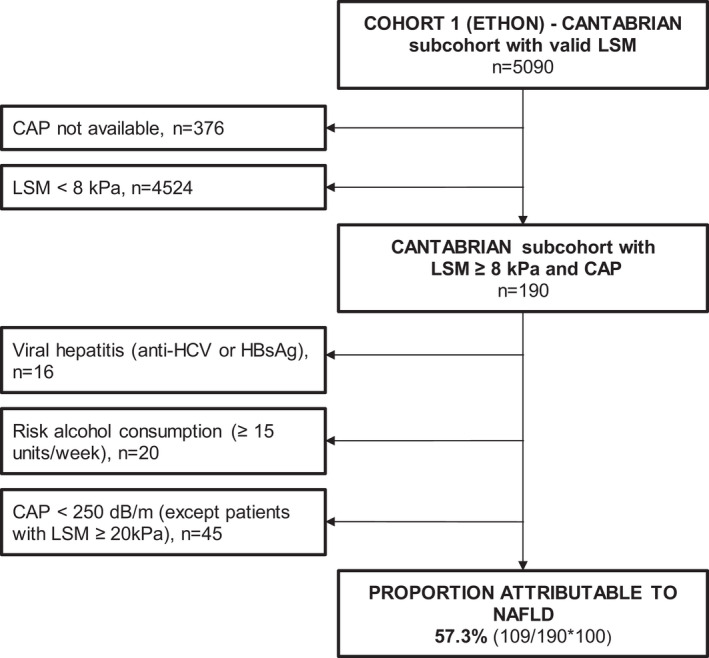
Flow chart for the calculation of the proportion attributable to non‐alcoholic fatty liver disease (NAFLD) in the population‐based cohort. CAP, controlled attenuation parameter; HBsAg, hepatitis B surface antigen; HCV, hepatitis C virus; LSM, liver stiffness measurements; NAFLD, non‐alcoholic fatty liver disease.

The general plan for the study (Figure 1) consisted of four steps:

- STEP 1: To estimate the prevalence and distribution of liver fibrosis through clinically relevant TE ranges in Spain's general population from a large population‐based cohort (ETHON).
- STEP 2: To estimate the prevalence of NAFLD within the subset of patients with liver stiffness measurements (LSM) ≥ 8 kPa in the same cohort. For this estimation, we used the subcohort from Cantabria, which was the largest subcohort (*N* = 5090) and contained 99.7% of all valid CAP measurements from the whole ETHON dataset. Patients with viral hepatitis (positive anti‐HCV or HBsAg), high‐risk alcohol consumption (≥ 15 units/week) assessed by AUDIT test and those with the absence of steatosis defined as CAP < 250 dB/m (except for patients with LSM ≥ 20 kPa in order to avoid “burn‐out” NASH cirrhosis exclusion) were excluded. The proportion attributable to NAFLD was calculated as the ratio of the remaining patients after these exclusions over the total of patients with LSM ≥ 8 kPa (Figure 2).
- STEP 3: To describe the distribution of the different liver fibrosis stages in a Spanish multi‐centre cohort of biopsy‐proven NASH with paired TE (LSM and CAP) data, contemporary to the population‐based cohort.
- STEP 4: To generate estimations of the real prevalence for the different NASH‐related fibrosis stages for Spain's general population by crossing the estimated prevalence of NAFLD drawn from ETHON cohort with the probability of each specific stage of fibrosis observed in the biopsy‐proven NASH cohort in patients with LSM ≥ 8 kPa.

As secondary outcome, using the same step by step approach detailed above, we aimed at describing prevalence estimates using the LSM 9.1 kPa cut‐off, which has been recently suggested as the most cost‐efficient LSM threshold for theoretical population‐level screening.^28^

The paper has been performed and written following the Strengthening the Reporting of Observational Studies in Epidemiology (STROBE) guidelines.^29^

### 2.2 Patients

The study integrates data from two large cohorts:

1. Population‐based cohort (ETHON cohort). The PREVHEP‐ETHON is a Spanish dataset composed of subjects aged 20–79 years selected from the general population of 18 primary care centres belonging to three university hospitals from 2015 to 2017. Participants were randomly selected and stratified by socioeconomical status, rural/urban setting and age. The geographical location of Santander, Madrid and Valencia (north, centre and east, respectively) determinates substantial differences in terms of weather, lifestyle and food habits, appropriately showing a real‐life picture of Spain at population level. Thus, this cohort is considered representative of Spain's general population and has served as a reference for other observational, cross‐sectional population‐based studies,^30^ even though the initial study was aimed to investigate the prevalence of HCV in the general population.^31^

This cohort was the basis for the estimation of TE values for the Spanish general population as well as for the estimation of the attributable causal weight of NAFLD within the range of LSM ≥8 kPa.

2 Biopsy‐proven NASH cohort. This dataset was a cross‐sectional, retrospective cohort of real clinical practice patients with biopsy‐proven NASH from two hospitals from the ETHON cohort (Marqués de Valdecilla, Cantabria and Puerta de Hierro, Madrid) and three additional tertiary centres (Virgen del Rocío, Seville; Clínico Universitario, Valladolid; and Vall d'Hebron, Barcelona). These centres had been collecting data prospectively from NAFLD patients for several studies and for Spain's NAFLD National Registry (Hepamet).^32^ Patient selection was based on what is estimated as current clinical practice in tertiary centres in Spain, where patients are referred to liver clinics based on a combination of altered liver function tests and/or finding of steatosis in liver ultrasound. Once in the clinics, patients with LSM < 8 kPa are considered low risk and are less likely to undergo liver biopsy, and in patients with LSM ≥ 8 kPa liver biopsy is individually decided for disease staging and for inclusion consideration in clinical trials. For the present study, we only included consecutive patients with paired data from liver biopsy and TE within a < 12‐month period obtained with Fibroscan devices equipped with M and XL probes, spanning from 2015 to 2020. Only liver samples clearly interpretable for the pathologists were included in the study.

The biopsy‐proven cohort was used to translate TE estimations for the general Spanish population into histology‐based estimations for NASH diagnosis and its different fibrosis stages.

### 2.3 Procedures

TE measurements, liver biopsy and histological evaluation were performed according to current standards.^33^, ^34^ A detailed description of procedures is provided in Supplementary Material.

### 2.4 Statistical analysis

For demographic extrapolations and comparisons with the Spanish general population, publicly available data from Instituto Nacional de Estadística (INE) were used. INE is a legally independent administrative institution which serves as main repository of demographic data for Spain, including key health indicators. Patients with non‐valid LSM and those with LSM < 8 kPa from both cohorts will be excluded from final analyses under the assumption that these patients would not be referred to tertiary centres to undergo liver biopsy in real clinical practice.

Categorical data are presented as number (percentage). Continuous data are presented as mean ± standard deviation and median (interquartile range). A *p* < .05 was considered statistically significant. Missing values were kept as missing, and no specific statistical procedures were used for imputations. Data were collected and edited using Microsoft Excel (version Microsoft Office Pro 2019). Statistical analyses were performed using PAWS Statistics (version 19.0; SPSS Inc., Hong Kong) software.

## 3 RESULTS

### 3.1 Patients characteristics from the population‐based transient elastography cohort (ETHON cohort)

From the complete ETHON dataset of 12 246 individuals, 806 (6.6%) were excluded because of non‐valid or indeterminate LSM.

For the 11 440 patients included in the analysis (Table 1) median age was 51 years, 58% were women and 88% were Caucasian. Median BMI was 26.1 kg/m^2^ and 60% were either overweight or obese. Diabetes prevalence was 13.5% and 5206 patients (53.6%) had arterial hypertension. Almost 16% of the population met NCEP‐ATP III criteria for metabolic syndrome.^35^

**TABLE 1:** Baseline characteristics of the patients from the general population (ETHON) cohort included in the analysis

General population cohort	*N* = 11 440
Age, years	51 (42–60)
Male, *n* (%)	4792 (41.9)
Geographic subcohort, *n* (%)	
Cantabria	5090 (44)
Madrid	4088 (36)
Valencia	2262 (20)
Caucasian, *n* (%)	10 058 (87.9)
Alcohol risk consumption, *n* (%)	442 (3.9)
Body mass index (BMI), kg/m^2^	26.1 (23.3–29.3)
Weight, *n* (%)	
Normal weight (<25 kg/m^2^)	3846 (40.4)
Overweight (≥25–<30 kg/m^2^)	3597 (37.8)
Obesity (≥30 kg/m^2^)	2075 (21.8)
Waist circumference, cm	90 (80–99)
Type 2 diabetes, *n* (%)	1540 (13.5)
Arterial hypertension, *n* (%)	5206 (53.6)
Dyslipidemia, *n* (%)	7418 (64.8)
Metabolic syndrome, *n* (%)	1764 (15.4)
Fasting glucose, (mg/dl)	86 (79–96)
Total cholesterol, (mg/dl)	197 (174–222)
HDL, (mg/dl)	57 (47–68)
LDL, (mg/dl)	113 (91–135)
Triglycerides, (mg/dl)	114 (78–172)
Creatinine, mg/dl	0.78 (0.67–0.91)
AST, (U/L)	22 (19–27)
ALT, (U/L)	20 (16–28)
ALP, (U/L)	68 (56–83)
GGT, (U/L)	20 (14–33)
Bilirubin, mg/dl	0.50 (0.40–0.65)
Albumin, g/dl	4.5 (4.3–4.6)
Platelets, x10E9/L	241 (205–282)
FIB–4 index	0.99 (0.72–1.36)
HBsAg positive, *n* (%)	90 (0.8)
Anti‐HCV positive, *n* (%)	143 (1.3)
Liver stiffness, (kPa)	4.5 (3.6–5.6)
CAP, (dB/m)^a^	247 (209–293)

*Note*: Risk alcohol consumption: ≥15 units of alcohol/week. Hypertension: ≥ 140/90 mmHg or requiring treatment; type 2 diabetes: as a fasting plasma glucose ≥126 mg/dl or a non‐fasting plasma glucose ≥180 mg/dl or requiring treatment.; dyslipidemia: serum triglycerides ≥150 mg/dl and/or total cholesterol >200 mg/dl, LDL >130 mg/dl, HDL < 40 mg/dl in men and < 50 mg/dl in women or requiring treatment.

^a^ Data from Cantabrian subcohort, *N* = 4714.

Subjects from Cantabria represented 44% of ETHON dataset. Within this subcohort, 23.4% were obese, 61.4% presented a BMI ≥ 25 kg/m^2^ and 20% met metabolic syndrome criteria. Median LSM and CAP values from Cantabria were 4.4 kPa and 247 dB/m respectively. As shown, baseline features between the Cantabrian subcohort and the whole ETHON dataset were comparable.

Metabolic estimates from ETHON cohort were consistent with available data from Spain's general population, where 22.9%, 13.8% and 42.6% have been estimated to present obesity, diabetes and arterial hypertension respectively.^36^, ^37^, ^38^, ^39^

### 3.2 STEP 1: TE ranges

The first step consisted of the estimation of LSM ranges in the general population. Out of the 11 440 individuals with reliable TE from ETHON cohort, 5.61% (95%CI 2.53–11.97) had LSM ≥ 8 kPa and 2.60% (95%CI 0.83–7.88) presented LSM ≥ 10 kPa (Table 2). Within the LSM ≥ 8 kPa subgroup, 53% (344/643*100) of patients fell in the 8–10 kPa range.

**TABLE 2:** Prevalence of different ranges of LSM in the general population (ETHON) cohort

LSM ranges	Patients, *n* (%)		
< 8 kPa	10 797 (94.39)	LSM ≥ 8 kPa, *n* (%)	
8–10 kPa	344 (3.00)	643 (5.61)	LSM ≥ 10 kPa, *n* (%)
10–15 kPa	185 (1.62)		299 (2.60)
15–20 kPa	36 (0.31)		
≥ 20 kPa	78 (0.68)		
Total	11 440 (100)		

Abbreviation: LSM, liver stiffness measurements.

### 3.3 STEP 2: Estimation of the prevalence attributable to NAFLD within the LSM ≥8 kPa subcohort

The second step was to estimate the aetiologic relative weight of NAFLD within the LSM ≥ 8 kPa population. For this specific purpose, as mentioned above, we used the subcohort from Cantabria, which was the largest subcohort and contained 4714/4728 (99.7%) CAP measurements from the whole ETHON dataset.

As seen in Figure 2, the proportion attributable to NAFLD was 57.3% (109/190*100), calculated as the ratio of the remaining subjects after exclusion of patients with viral hepatitis, high‐risk alcohol consumption and those with the absence of steatosis assessed by CAP, over the total of patients with LSM ≥ 8 kPa and available CAP. Thus, the prevalence of individuals with LSM ≥ 8 kPa because of NAFLD in the general population was estimated to be 3.21% (5.61*0.573).

### 3.4 Patient characteristics from the biopsy‐proven NASH cohort

The real clinical practice dataset was composed of 501 consecutive patients with histological confirmation of NASH and paired TE measurements from five tertiary hospitals in Spain. The main clinical features were usual in European NASH cohorts. Median age was 59 years and 57% were males. Median BMI was 32 kg/m^2^, 66% of patients had obesity and 44% type 2 diabetes. Median time between TE and liver biopsy was 2.29 months (IQR 1.12–4.41) and only 12.5% (63/501) of patients presented a time interval > 6 months between procedures. A detailed description of the baseline characteristics from the biopsy‐proven NASH cohort and the comparison between both study cohorts are provided in Table S1.

### 3.5 STEP 3: Description of the distribution of histological NASH‐related fibrosis according to different LSM ranges

Among the 501 patients of the whole biopsy‐proven NASH cohort, 112 patients had LSM < 8 kPa. Among these patients, 92 (82%) were F0‐1, 15 presented F2, only 5 were F3 and no patients had cirrhosis on histology.

**FIGURE 3:**
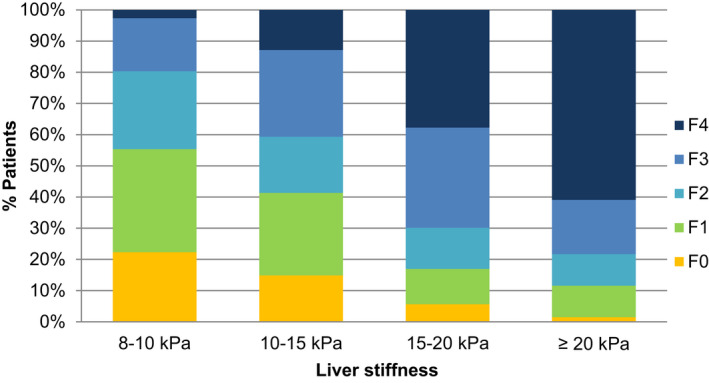
Distribution of liver fibrosis within increasing ranges of LSM in the biopsy‐proven cohort of patients with non‐alcoholic steatohepatitis (NASH)

The distribution of fibrosis by different LSM ranges for the remaining 389 patients are shown in Figure 3 and Table 3. Among patients with LSM ≥ 8 kPa, 37% (143/389) had F0‐1 and, for those patients with LSM ≥ 10 kPa, as much as 44% (123/277) did not show advanced fibrosis (F3‐4) at histology. As seen, the proportion of patients with advanced fibrosis increased at each LSM interval, but even at the highest interval (LSM ≥ 20 kPa), there was a substantial proportion of patients without cirrhosis (39%).

**TABLE 3:** Distribution of histological liver fibrosis according to LSM in the biopsy‐proven NASH cohort

	LSM ranges, *n* (%)					
Fibrosis	8–10 kPa	10–15 kPa	15–20 kPa	≥ 20 kPa	LSM ≥ 8 kPa	LSM ≥ 10 kPa
F0	25 (22.32)	23 (14.84)	3 (5.66)	1 (1.44)	52 (13.37)	27 (9.75)
F1	37 (33.04)	41 (26.45)	6 (11.32)	7 (10.15)	91 (23.39)	54 (19.49)
F2	28 (25.00)	28 (18.07)	7 (13.20)	7 (10.15)	70 (18.00)	42 (15.16)
F3	19 (16.96)	43 (27.74)	17 (32.08)	12 (17.39)	91 (23.39)	72 (26.00)
F4	3 (2.68)	20 (12.90)	20 (37.74)	42 (60.87)	85 (21.85)	82 (29.60)
Total	112 (100)	155 (100)	53 (100)	69 (100)	389 (100)	277 (100)

Abbreviation: LSM, liver stiffness measurements.

Regarding liver biopsy quality parameters, median tissue length among patients with LSM ≥ 8 kPa was 23.0 mm (IQR 19.0–28.0), and only 5.1% of samples were fragmented into more than two pieces. Of note, the subset of patients within the highest LSM interval (≥ 20 kPa) showed similar quality data (21.5 mm median size ‐IQR 17.0–27.0‐ and 4.5% fragmentation respectively).

### 3.6 STEP 4: Prevalence estimation of NASH‐related fibrosis stages for Spain's general population (2015–2020)

The main study outcome was calculated by merging the main results from the two cohorts. The prevalence for each NASH‐related fibrosis stage in Spain's general population was estimated by crossing the estimated prevalence of NAFLD with LSM ≥ 8 kPa (3.21%) drawn from ETHON dataset with the different fibrosis stages probabilities observed in the biopsy‐proven NASH cohort (detailed in Table 3—LSM ≥ 8 kPa column). The final calculations from our study are shown in Table 4 (i.e., prevalence of F2 fibrosis stage resulted in 0.58% = 3.21*0.18). Finally, the estimated prevalence of NASH with significant fibrosis F2‐4 in Spain was 2.03 (95% CI 0.56–7.05), 1.33 (95% CI 0.29–5.98) of the Spanish population aged 20–79 years presented NASH F2‐3 and 0.70 (95% CI 0.10–4.95) was estimated to have NASH cirrhosis.

**TABLE 4:** Prevalence estimation of the different NASH fibrosis stages in Spain's general population

	Nash fibrosis prevalence (%)			
Fibrosis	LSM ≥ 8 kPa (95%CI)	Significant fibrosis (F2‐F4)	Intermediate stages (F2‐F3)	Cirrhosis (F4)
F0	0.43 (0.04–4.49)			
F1	0.75 (0.11–5.04)			
F2	0.58 (0.07–4.75)	2.03 (0.56–7.05)	1.33 (0.29–5.98)	
F3	0.75 (0.11–5.04)			
F4	0.70 (0.10–4.95)			0.70 (0.10–4.95)
Total	3.21 (1.13–8.75)			

Abbreviations: CI, confidence interval; LSM, liver stiffness measurements; NASH, non‐alcoholic steatohepatitis.

Additional estimations using the LSM ≥ 10 kPa threshold and alternative approximations to the proportion attributable to NAFLD using more or less conservative CAP thresholds (275 or 220 dB/m, respectively) are provided in Tables S2–S5.

### 3.7 Prevalence estimates with LSM 9.1 kPa threshold

The LSM ≥ 9.1 kPa threshold has been suggested as the most cost‐effective cut‐off for a theoretical TE‐based screening programme of liver fibrosis in populations with lower prevalence of risk alcohol consumption, such as Spain.^28^ Applying the same methodology used for the 8 kPa threshold we found that in ETHON cohort, prevalence of LSM ≥ 9.1 kPa was 3.36%. In our biopsy‐proven cohort, 66.5% (223/335) of patients had significant fibrosis (F2‐4) and 49.8% (167/335) presented advanced fibrosis (F3‐4). The proportion attributable to NAFLD resulted in 55.2% of cases of all Spain's population with LSM ≥ 9.1 kPa, and thus 1.85% of Spain's population would be assumed to have NAFLD, with 1.10% estimated to have F2‐4.

Of note, raising the threshold from 8.0 to 9.1 kPa in a hypothetical screening plan would represent a 39.9% relative reduction in the proportion of patients that would be targeted. However, according to our biopsy‐proven cohort, in the subcohort of 54 patients with LSM 8–9.1 kPa, 9 (16.6%) had advanced fibrosis. The advantages and risks of using the 9.1 kPa threshold should be weighted in dedicated cost‐effective studies.

## 4 DISCUSSION

In the present study, we provide an updated estimation of the prevalence of NASH‐related fibrosis in Spain's general population, by combining TE data from a large population‐based screening cohort, and histological information from a contemporary multi‐centre cohort of biopsy‐proven NASH from Spain. We observed that 5.61% of the general population had LSM ≥ 8 kPa, 57.3% of which (3.21%) was attributable to NAFLD. By crossing these estimates with the corresponding probabilities for the different fibrosis stages by LSM intervals drawn from the biopsy‐proven cohort, we estimated that the current prevalence of NASH with significant fibrosis in Spain was 2.03% (1.33% NASH F2‐3 and 0.70% NASH cirrhosis).

The estimates on NASH with significant fibrosis from our study are lower than those provided in previous epidemiological reports from other European cohorts,^28^, ^40^, ^41^, ^42^ even though the prevalence of LSM ≥ 8 kPa in the ETHON population is almost the same. The most likely reason for these differences stems from the fact that fibrosis estimations in those studies were fundamentally TE‐based (backed up by the absence of liver biopsies or a relatively small number of them) and, in the present study, TE estimations were back‐tested against a large contemporary histological dataset, which clearly suggests that current TE‐based definitions are overestimating the prevalence of fibrosis in NASH. In fact, 37% of the patients in our study with LSM ≥ 8 kPa did not have “significant fibrosis,” and 44% of patients with LSM ≥ 10 kPa did not have “advanced fibrosis” at histology. A recent paper,^28^ integrating population‐based TE data with histological data from 6 independent cohorts, with 6300 patients and 350 biopsies, showed an estimated prevalence of F2‐4 in the general population (all aetiologies combined) of 3.9%. In our study, there were more than 12 000 patients in the population‐based cohort and more than 500 biopsies, which served at narrowing confidence intervals for the different degrees of fibrosis that could be expected at each TE interval, providing more precise references for the population‐based estimates. As a practical consequence, we firmly believe that current TE‐based definitions of “significant” and “advanced” fibrosis (LSM ≥ 8 kPa and 10 kPa, respectively) should be avoided, since they provide unrealistic overestimations of the actual prevalence of the corresponding fibrosis stages.

The second reason for the different prevalence of NASH F2‐4 is the more comprehensive attribution of aetiology made in our study, where we estimated that approximately 60% of patients with LSM ≥ 8 kPa from the general population had NAFLD. In the paper by Caballeria et al.,^40^ attribution of NAFLD aetiology was made by exclusion of with viral hepatitis and patients with high‐risk alcohol consumption, resulting in a remarkably high aetiologic weight of NAFLD (near 90%). On the other hand, in the other two large European studies,^41^, ^42^ NAFLD was suspected basically on clinical grounds, yielding a much lower estimated prevalence (32%–42% for the whole LSM spectrum). By contrast, to estimate the proportion attributable to NAFLD we added the use of CAP in nearly 5000 patients from a representative subcohort, enabling the extrapolation of the results to the whole dataset and, thus, to the general population. We believe that the combination of CAP along clinical and laboratory values in the present study likely provides the most accurate estimation for the attribution of aetiology in population‐based studies to date.

The study has nonetheless some limitations that should be taken into account when interpreting the transferability of our results to real practice decisions. The main limitation is the risk of overestimation of the prevalence of F2‐4 stages, as consequence of unavoidable referral and selection bias. It has been consistently shown that NASH prevalence is overestimated in tertiary settings.^2^ Local initiatives for structured early detection and referral have been developed in recent years in the biopsy‐proven cohort centres and may have helped at buffering that risk. However, the efficiency of systematic referral is still suboptimal, and thus mitigation of referral bias is probably modest. The risk of selection bias is also unavoidable in this kind of retrospective studies since decision for biopsy was not standardized and could have been influenced by other criteria different from LSM. However, it should be noted that current practice in all participating centres is quite homogenous and representative of what is done in daily clinical practice in Spain. In fact, the 2 main centres recruiting for the ETHON cohort were also part of the contemporary biopsy‐proven cohort (Cantabria, Madrid). In any case, the ideal approach to minimize these biases would be the implementation of prospective large‐scale screening programmes, with per‐protocol liver biopsies above a pre‐specified LSM threshold. Such a study is already ongoing (LiverScreen, H2020‐EU ID 847989), but results of such large efforts will take long. Thus, until this sort of large‐scale prospective data becomes available, the estimations provided in the present study remain as the most accurate approximations to the prevalence of NASH with significant fibrosis in a European population.

Other limitations should be considered. Biopsy reading was not centralized and could be subject of interobserver variability, although we believe this is a reflection of what should be expected in real‐life situations. We acknowledge that the attribution of the relative weight of NAFLD as aetiology was imperfect. The choice of the CAP threshold was arbitrary, since there is no clear consensus in the literature and there are no clear published reports on the specificity and positive predictive values of CAP estimates for patients with higher LSM.^10^ It can be also argued about the positive predictive value of 250 dB/m CAP threshold to identify steatosis at general population level. This issue can be partially mitigated in two ways. First, by adjusting CAP values according to influencing covariables, such as type‐2 diabetes, BMI and liver aetiology.^18^ Second, as provided in the present study, by generating alternative estimations using different CAP cutoffs with greater or lesser sensitivity (including the 275 dB/m threshold suggested in the latest clinical practice guideline).^17^ Moreover, the estimation on the relative weight of NAFLD would require adjustment by the alcoholic liver disease burden in each country. There will be also patients with other liver aetiologies, although their reported frequencies in primary care series are very low (<2%) and should not distort significantly large‐scale epidemiological estimations.^43^ Finally, the ethnic background in our study is predominantly Caucasian, so extrapolation to populations with more diverse ethnicities should be done with caution.

In conclusion, our large‐scale epidemiological study, merging population‐based TE data with histological data from real‐world practices, provides accurate and updated estimates of the current prevalence of NASH‐related fibrosis in Spain, with a special focus on those stages that should be the target of early detection and referral policies to identify high‐risk patients who will benefit more from the pharmacological therapies under development.

Until large‐scale prospective epidemiological data with biopsy confirmation becomes available, our results could serve as reference points for both assessing the current burden of disease and for modelling changes in time, so health policies can be designed and adapted accordingly, and besides for dimensioning the therapeutic efforts that will be required as the time when we have effective treatments to treat NASH approaches.

## FUNDING INFORMATION

The work was independent of all funding. JRE is a PhD student at Universitat Autònoma de Barcelona, Spain. JC is a receptor of a grant of Fondo Investigaciones Sanitarias (FIS): PI18/01304. Immunomediated Nonalcoholic SteaTohepatItis; prevalence and CharacTerization. INSTInCT.

## CONFLICT OF INTEREST

JLC received consulting and lecture fees for Intercept, Echosens and Gilead Sciences. JMP received consulting, educational or research grants for Gilead, NovoNordisk, Novartis, Boehringer‐Ingelheim, BMS, Pfizer, Accelerate, Astellas, ViiV, Janssen, MSD, Abbie. MRG received consulting fees from Alpha‐sigma, Allergan, BMS, Boehringer‐Ingelheim, Gilead, Intercept, Kaleido, MSD, Pfizer, Prosciento, Shionogi, Sobi, Zydus and research Grants: Gilead, Intercept. SA changed affiliations after finalizing the manuscript and its submission to the journal. Boehringer Ingelheim did not fund the study and did not have any role in the study design, analysis or interpretation of the data, writing or revision of the manuscript or the decision to submit the manuscript for publication. Other potential conflicts of interest SA: consulting fees from Boehringer Ingelheim, Ferrer, Gilead, Intercept, IQVIA, Novartis, Pfizer; speaking fees from Allergan, Gilead, MSD and Novartis; travel expenses from Gilead, MSD, Janssen, Genfit, Bayer and Ferring; grant support from Gilead. JC received consulting fees from Gilead, Intercept, MSD, Abbie; grant support from Gilead, MSD.

## ETHICS STATEMENT

The study protocol was approved by Vall d'Hebron Ethics Committee for Clinical Research (PR[AG]655/2020) and conformed to the ethical guidelines of the 1975 Declaration of Helsinki.

## PATIENT CONSENT FOR PUBLICATION

Not required.

## PERMISSION TO REPRODUCE MATERIAL FROM OTHER SOURCES

The material in this paper is original and does not come from other sources.

## Supporting information


**Appendix S1** xxx
