# Supplementary material for: Prevalence estimation of significant fibrosis because of NASH in Spain combining transient elastography and histology
Source: Liver Int. 2022 Jun 7;42(8):1783–92. doi: 10.1111/liv.15323 (PMC9541569; doi:10.1111/liv.15323)
Supplement: Supplementary file 1 — Appendix S1 xxx [file LIV-42-1783-s001.docx]

**Supplementary material**

**Procedures**

-Transient elastography: LSM and CAP values were obtained from all participants at their corresponding centres using Fibroscan 502 Touch devices (Echosens, Paris, France) equipped with M and XL probes. All measurements were performed by a specialized health-care professional experienced with the procedure (> 500 examinations), using the probe recommended by the device for each patient. TE measurements were performed under usual and manufacturer standards. A LSM was considered reliable if an interquartile range/median (IQR/M) ratio < 0.30 was achieved, and only examinations with at least 10 individual measurements were deemed valid. The CAP algorithm calculates the attenuation of the ultrasound signal during the TE examination and correlates with the histological degree of steatosis.

-Liver biopsy and histopathological evaluation: In each centre, indication for percutaneous liver biopsy was made upon TE and clinical criteria, as detailed in the main text. Biopsies were performed, fixed and stained according to the usual standard procedures and analyzed at each centre by a senior pathologist specialized in liver pathology. NASH features and fibrosis stage were described, and NASH activity score was calculated using the NASH clinical research network (CRN) scoring system. NASH was diagnosed using the “fatty liver: inhibition of progression” definition (concurrent presence of steatosis, hepatocyte ballooning, and lobular inflammation with at least 1 point for each category). Significant fibrosis was defined as F≥2, advanced fibrosis as F≥3, and cirrhosis as F4.

**Supplementary Table 1. Baseline characteristics of the patients included in the biopsy-proven NASH cohort and comparison between the two study cohorts.**

| **Variables** | **General population cohort (ETHON) N=11440** | **Biopsy-proven NASH cohort N=501** | **p value** |
| --- | --- | --- | --- |
| Age, years | 51 (42-60) | 59 (50-65) | <0.001 |
| Male, n (%) | 4792 (41.9) | 283 (56.5) | <0.001 |
| Geographic subcohort, n (%) |  |  |  |
| Cantabria | 5090 (44) |  |  |
| Madrid | 4088 (36) |  |  |
| Valencia | 2262 (20) |  |  |
| Centre, n (%) |  |  |  |
| Marqués Valdecilla, Santander |  | 200 (40) |  |
| Vall d'Hebron, Barcelona |  | 135 (27) |  |
| Puerta de Hierro, Madrid |  | 100 (20) |  |
| Clínico, Valladolid |  | 45 (9) |  |
| Virgen del Rocío, Seville |  | 21 (4) |  |
| Caucasian, n (%) | 10058 (87.9) |  |  |
| Body mass index (BMI), kg/m^2^ | 26.1 (23.3-29.3) | 32.3 (28.4-36.5) | <0.001 |
| Weight, n (%) |  |  | 0.20 |
| Normal weight (<25 kg/m^2^) | 3846 (40.4) | 40 (8.0) | <0.001 |
| Overweight (≥25 -<30 kg/m^2^) | 3597 (37.8) | 129 (25.7) | <0.001 |
| Obesity (≥30 kg/m^2^) | 2075 (21.8) | 332 (66.3) | <0.001 |
| Waist circumference, cm | 90 (80-99) | 108 (99-118) | <0.001 |
| Type 2 diabetes, n (%) | 1540 (13.5) | 222 (44.3) | <0.001 |
| Arterial hypertension, n (%) | 5206 (53.6) | 278 (55.5) | 0.20 |
| Dyslipidemia, n (%) | 7418 (64.8) | 405 (80.8) | <0.001 |
| Metabolic syndrome, n (%) | 1764 (15.4) | 313 (62.5) | <0.001 |
| Fasting glucose, (mg/dl) | 86 (79-96) | 106 (92-127) | <0.001 |
| Total cholesterol, (mg/dL) | 197 (174-222) | 186 (163-220) | <0.001 |
| HDL, (mg/dL) | 57 (47-68) | 45 (39-56) | <0.001 |
| LDL, (mg/dL) | 113 (91-135) | 110 (90-140) | 0.91 |
| Triglycerides, (mg/dL) | 114 (78-172) | 145 (107-199) | <0.001 |
| Creatinine, mg/dl | 0.78 (0.67-0.91) | 0.78 (0.65-0.92) | 0.46 |
| AST, (U/L) | 22 (19-27) | 36 (26-55) | <0.001 |
| ALT, (U/L) | 20 (16-28) | 47 (31-75) | <0.001 |
| ALP, (U/L) | 68 (56-83) | 83 (65-106) | <0.001 |
| GGT, (U/L) | 20 (14-33) | 62 (37-137) | <0.001 |
| Bilirubin, mg/dl | 0.50 (0.40-0.65) | 0.60 (0.4-0.8) | <0.001 |
| Albumin, g/dl | 4.5 (4.3-4.6) | 4.4 (4.2-4.6) | 0.09 |
| Platelets, x10E9/L | 241 (205-282) | 222 (180-264) | <0.001 |
| FIB - 4 index | 0.99 (0.72-1.36) | 1.43 (0.99-2.10) | <0.001 |
| Liver stiffness, (kPa) | 4.5 (3.6-5.6) | 10.5 (8.3-14.8) | <0.001 |
| CAP, (dB/m) † | 247 (209-293) | 325 (291-363) | <0.001 |
| Alcohol risk consumption, n (%) | 442 (3.9) |  |  |
| Anti HCV positive, n (%) | 143 (1.3) |  |  |
| HBsAg positive, n (%) | 90 (0.8) |  |  |
| Time from TE to liver biopsy, months |  | 2.29 (1.12-4.41) |  |
| Liver sample size, mm ‡ |  | 23.0 (19.0-28.0) |  |

†Data from Cantabrian subcohort, N=4714.
‡ Data from patients with LSM ≥ 8 kPa, N=389
Risk alcohol consumption: ≥21/≥15 units of alcohol/week (men/women). Hypertension: ≥ 140/90 mmHg or requiring treatment; type 2 diabetes: as a fasting plasma glucose ≥ 126 mg/dL or a non-fasting plasma glucose ≥ 180 mg/dL or requiring treatment.; dyslipidemia: serum triglycerides ≥150 mg/dL and/or total cholesterol >200 mg/dl, LDL >130 mg/dl, HDL<40 mg/dL in men and <50 mg/dL in women or requiring treatment.

Abbreviations: ALP, alkaline phosphatase; ALT, alanine aminotransferase; AST, aspartate aminotransferase; BMI, body mass index; CAP, controlled attenuation parameter GGT, gamma-glutamyl transferase; HBsAg; hepatitis B surface antigen; HCV, hepatitis C virus; HDL, high density lipoprotein; LDL, low density lipoprotein.

**Supplementary Table 2. Estimation of the NAFLD prevalence in general population using different LSM and CAP thresholds.**

|  | **CAP ≥ 220 dB/m** | | **CAP** **≥ 250 dB/m** | | **CAP** **≥ 275 dB/m** | |
| --- | --- | --- | --- | --- | --- | --- |
| **LSM RANGE** | **LSM ≥ 8 kPa (95%CI)** | **LSM ≥ 10 kPa (95%CI)** | **LSM ≥ 8 kPa (95%CI)** | **LSM ≥ 10 kPa (95%CI)** | **LSM ≥ 8 kPa (95%CI)** | **LSM ≥ 10 kPa (95%CI)** |
| **TOTAL POPULATION (%)** | 5.61 (2.5-11.9) | 2.60 (0.83-7.88) | 5.61 (2.5-11.9) | 2.60 (0.83-7.88) | 5.61 (2.5-11.9) | 2.60 (0.83-7.88) |
| **ATRIBUTTABLE TO NAFLD (%)** | 63.1 | 60.6 | 57.3 | 57.4 | 49.5 | 50.5 |
| **POPULATION WITH NAFLD (%)** | 3.53 (1.31-9.19) | 1.57 (0.37-6.35) | 3.21 (1.13-8.75) | 1.49 (0.34-6.23) | 2.77 (0.91-8.12) | 1.31 (0.28-5.94) |

CAP controlled attenuation parameter; CI, confidence interval; LSM, liver stiffness measurements.

**Supplementary Table 3. Estimation of the prevalence of NASH-related fibrosis stages in Spain’s general population using the 10 kPa LSM threshold.**

|  | **NASH FIBROSIS PREVALENCE (%)** | | | |
| --- | --- | --- | --- | --- |
| **FIBROSIS** | **LSM ≥ 10 kPa (95%CI)** | **SIGNIFICANT FIBROSIS (F2-4)** | **INTERMEDIATE STAGES (F2-3)** | **CIRRHOSIS (F4)** |
| **F0** | 0.14 (0.01-3.96) |  |  |  |
| **F1** | 0.29 (0.02-4.24) |  |  |  |
| **F2** | 0.23 (0.01-4.13) | 1.06 (0.20-5.55) | 0.62 (0.08-4.82) |  |
| **F3** | 0.39 (0.03-4.42) |  |  |  |
| **F4** | 0.44 (0.04-4.51) |  |  | 0.44 (0.04-4.51) |
| **TOTAL** | **1.49 (0.34-6.23)** |  |  |  |

CI, confidence interval; LSM; liver stiffness measurements; NASH, non-alcoholic steatohepatitis.

**Supplementary Table 4. Estimation of the prevalence of NASH-related fibrosis stages in Spain’s general population using the 8 and 10 kPa LSM thresholds when using the CAP 220 dB/m threshold.**

| **NASH F PREVALENCE (%) when using CAP ≥ 220 dB/m** | | |
| --- | --- | --- |
|  | **LSM ≥ 8 kPa (95%CI)** | **LSM ≥ 10 kPa (95%CI)** |
| **SIGNIFICANT FIBROSIS (F2-4)** | 2.23 (0.65-7.34) | 1.11 (0.21-5.63) |
| **INTERMEDIATE STAGES (F2-3)** | 1.46 (0.33-6.18) | 0.65 (0.08-4.87) |
| **CIRRHOSIS (F4)** | 0.77 (0.11-5.07) | 0.46 (0.04-4.54) |

.

CAP controlled attenuation parameter; CI, confidence interval; LSM; liver stiffness measurements; NASH, non-alcoholic steatohepatitis

**Supplementary Table 5. Estimation of the prevalence of NASH-related fibrosis stages in Spain’s general population using the 8 and 10 kPa LSM thresholds when using the CAP 275 dB/m threshold.**

| **NASH F PREVALENCE (%) when using CAP ≥ 275 dB/m** | | |
| --- | --- | --- |
|  | **LSM ≥ 8 kPa (95%CI)** | **LSM ≥ 10 kPa (95%CI)** |
| **SIGNIFICANT FIBROSIS (F2-4)** | 1.75 (0.45-6.62) | 0.93 (0.16-5.33) |
| **INTERMEDIATE STAGES (F2-3)** | 1.15 (0.22-5.69) | 0.54 (0.06-4.68) |
| **CIRRHOSIS (F4)** | 0.60 (0.07-4.78) | 0.39 (0.03-4.42) |

CAP controlled attenuation parameter; CI, confidence interval; LSM; liver stiffness measurements; NASH, non-alcoholic steatohepatitis
